# Security-Enhanced 3D Data Encryption Using a Degradable pH-Responsive Hydrogel

**DOI:** 10.3390/nano11071744

**Published:** 2021-07-01

**Authors:** Hongjing Wen, Bin Wang, Hongbo Zhu, Shiyu Wu, Xiaoxuan Xu, Xiangping Li, Yaoyu Cao

**Affiliations:** 1 The Key Laboratory of Weak-Light Nonlinear Photonics, Ministry of Education, School of Physics, Nankai University, Tianjin 300071, China; 1120180052@mail.nankai.edu.cn (H.W.); wb@nankai.edu.cn (B.W.); 2 Guangdong Provincial Key Laboratory of Optical Fiber Sensing and Communications, Institute of Photonics Technology, Jinan University, Guangzhou 511443, China; zhb975667@163.com (H.Z.); wushiyu001@stu2018.jnu.edu.cn (S.W.); xiangpingli@jnu.edu.cn (X.L.)

**Keywords:** degradable, pH-responsive hydrogel, data encryption

## Abstract

Based on degradable pH-responsive hydrogel, we report on an enhanced three-dimensional data encryption security technique in which a pH value is used for information manipulation. Featuring three types of states upon the pH value variation, namely, shrinkage, expansion and degradation, the hydrogel renders a limited pH value window as the “key” for information decryption. The pH-dependent shrinkage-to-expansion conversion of the hydrogel leads to a threshold pH value for retrieving the recorded data, whilst the degradability of the hydrogel, which can be tuned by adjusting the composition ratio of PEGDA/AAc, gives rise to a second threshold pH value for irreversibly sabotaging the retrieved data. Pre-doping silver ions in the hydrogel facilitates explicit recording and reading of binary data in forms of three-dimensional silver patterns through photoreduction and scattering, respectively, with a femtosecond laser. By accurately matching the vertical spacing of the encoded silver nanopatterns with the diffraction-limited focal depth of the decryption microscope, we can tune the pH value to encrypt and retrieve information recorded in layers and set a critical pH value to smash encoded information, which proves a highly secured 3D data encoding protocol. This strategy can effectively enrich data encryption techniques, vastly enhancing data security within unattained chemical dimensions.

## 1. Introduction

With the development of optical storage technology, the demand to protect data from intentional or unintentional disclosure has become increasingly high [1,2]. Thus far, several optical data encryption techniques have been developed, and their applications in document security [3], data storage [4,5], anti-counterfeiting [6,7], and other aspects have been continuously explored. For these applications, information stored in the storage medium can be read repeatedly through the effective key, but cannot be protected from attacks of invalid keys. For example, encrypted patterns can be broken through repeatedly stimulating a reusable data carrier medium [8] or iteratively reading through specific optical excitation conditions (such as the phase or polarization properties of light) [9,10,11,12] for decoding pre-stored data. The reversible and lossless nature of these encryption methods increases the possibility of secondary information leakage that has not been properly addressed [13]. The invalid key has shown no adverse effect upon information and its carrier in previous reports; therefore, there remain challenges in further enhancing data security by developing new encryption approaches, in which false keys may directly affect the existence of information before data reading.

In recent years, functional materials have received great attention for their potential roles as promising security-strengthened data storage media [14,15,16]. Taking advantage of the unique reactivities of these functional materials, various data encryption platforms have been developed, including carbon dot composites [17,18,19,20], AIE Polymers [21], amorphous organic materials [22], oxazolidine-functionalized nanoparticles [23], tetraphenylethylene@graphene oxide [24], smart hydrogel materials [25,26,27,28,29], etc. Among these materials, hydrogel with unique three-dimensional porous structures [30,31,32] exhibits outstanding versality and feasibility for carrying information and manipulating multidimensional data. In our previous study, the data encryption dimension was extended to chemical response-based decoding by utilizing pH-responsive hydrogel, in which encrypted information can be safely decrypted by applying correct pH values [33]. Nevertheless, because an invalid pH key shows no damage to the encoded information before regaining data, the previous protocol allows repeated decryption tests at different pH values until the effective information is retrieved, leading to a high risk of information leakage.

**Scheme 1:**
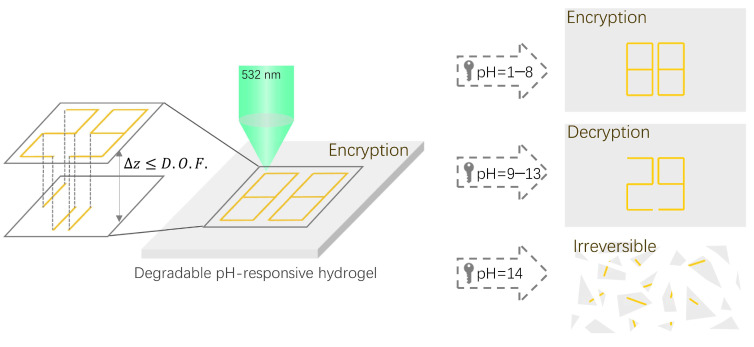
Schematic illustration of degradable pH-responsive hydrogel for security-enhanced 3D data encryption. Information recording is shown in the left figure; the double-layer structure written by the 532 nm femtosecond laser has a layer spacing for ∆*z* (∆*z* ≤ D.O.F., D.O.F. represents the depth of focus of the decryption system). The right figure is the schematic diagram of information reading. Different pH values are applied to the recording medium with encrypted information for data decryption, and three information states can be obtained, namely, encryption, decryption and irreversible.

Here, we demonstrate a security-enhanced 3D data encryption technique using a degradable pH-responsive composite hydrogel. The degradability of hydrogel makes it possible for information and its carrier to be destroyed by the invalid key. We developed a regulation protocol for controlling the pH value-dependent degradability by adjusting the composition of two polymeric components for the hydrogel design. The degradable hydrogel was prepared by using an acrylic acid (AAc) monomer and polyethylene glycol diacrylate (PEGDA) crosslinker [34]. We have shown that the PEGDA/AAc composite hydrogel exhibits obvious threshold stimuli-response behavior, i.e., hydrogel expansion can only occur under a specific pH value. By adjusting the composition mass ratio of PEGDA/AAc, degradation of the hydrogel was realized at specific alkali conditions, which led to a second threshold pH value. For data recording and reading, the hydrogel was pre-doped with a diamine silver complex ([Ag(NH_3_)_2_]^+^). Under the irradiation of femtosecond laser beam, the [Ag(NH_3_)_2_]^+^ complex ions in the vicinity of the tight focus obtained enough energy through a two-photon absorption process and reacted to form Ag nanopatterns attached to the gel network [35]. Notably, silver nanopatterns reveal strong surface plasmon-scattering effects similarly to noble nanometals [36], making them a gratifying candidate for optical data storage and data encryption. Resorting to volumetric resolution of the focused laser beam applied in information recording, we achieved 3D data encryption within the hydrogel for the first time. As shown in the Scheme 1, a plasmonic structure composed of silver nanolines was stored in a two-layered form within a degradable pH-responsive hydrogel by programmed laser beam positioning. The vertical layer-to-layer distance (∆*z*) was precisely controlled to approximate the depth of focus (*D.O.F.*) of the decoding dark-field microscopy system during the recording information process. Once the data are encoded, the resolving power of a microscope can protect the encrypted information from being regained without decryption keys. The information reading process is shown on the right. When different pH keys are applied to an information system, three information states can be obtained, namely, encryption, decryption and irreversible. The encrypted data can be clearly decoded at a specified pH value range, whereas the vertical spacing of the encoded layers is larger than the focal depth of the microscopic system (Δz>D.O.F.) for information decryption. Furthermore, when the invalid key with other pH values is applied, the encrypted message “29” cannot be read. The invalid key pH = 14 acts as a bomb hidden in all possible keys, and once touched, the information storage system will collapse irreversibly. In this case, the encrypted information is encrypted permanently.

## 2. Materials and Methods

### 2.1. Materials

Acrylic acid (AAc) (Shanghai Macklin Biochemical Co., Ltd., Shanghai, China), poly(ethylene glycol) diacrylate (PEGDA) (Aladdin, Shanghai, China), azobisisobutyronitrile (AIBN) (Shanghai Macklin Biochemical Co., Ltd., Shanghai, China) and dimethyl sulfoxide (DMSO, ST038) (Beyotime, Shanghai, China) were used as the raw materials to prepare the degradable pH-responsive hydrogel. n-Decanoylsarcosine sodium (NDSS) [37] was purchased from Tokyo Chemical Industry (Tokyo, Japan). Sodium hydroxide (NaOH) and nitric acid (HNO_3_, G.R., Guangdong Guangshi Regent Technology Co., Ltd., Guangzhou, China) were purchased from Sigma Aldrich. During the whole study, aqueous solutions were prepared using ultrapure water (Smart2Pure, Thermo Fisher, Waltham, MA, USA). All chemical reagents were of analytical grade.

### 2.2. Preparation of the PEGDA/AAc Composite Hydrogel

The degradable pH-responsive hydrogel was prepared with a four-step procedure. Firstly, a homogeneous solution was configured by mixing the monomer AAc (0.7 g), the crosslinker PEGDA (0.05 g), the initiator AIBN (0.05 g) and DMSO (1.0 g), and stirring for 20 min. Secondly, the gel solution was cured under the irradiation of ultraviolet light (395 nm, 5 min). Then, the hydrogel was immersed with deionized water to remove the residual DMSO. Finally, the resulting hydrogel was carefully cut into squares with 1 mm thickness, and immersed in fresh NDSS/diamine silver solution (C_Ag+_: 0.01mol/L, C_NDSS_: 0.01 mol/L) for 1 min to allow the silver ions to diffuse into the whole hydrogel network.

### 2.3. Acid/Alkali Treatments

NaOH and HNO_3_ were used to produce the dilute aqueous solutions with different pH values. The pH sensitivity of the degradable composite hydrogel was investigated by immersing square-shaped gels into different pH solutions (50 °C) for about 30 min. Then, the side lengths of gels were measured after reaching the equilibrium state. The scaling factor *r* of the geometrical variation of the hydrogel was defined as follows:

(1) $$r=\frac{l}{l_{0}}\;,$$

where $l_{0}$ (5 mm) represents the side length of the square original hydrogel which is completely immersed in deionized water without being stimulated by pH solution, and l represents the side length of the gel after soaking in a pH solution for 30 min.

### 2.4. Data Inscription

The hydrogel was pre-doped with diamine silver complex ([Ag(NH_3_)_2_]^+^). Under the irradiation of a femtosecond laser beam, the [Ag(NH_3_)_2_]^+^ complex ions in the vicinity of the tight focus obtained enough energy through a two-photon absorption process and reacted to form Ag nanopatterns attached to the gel network [38]. The PEGDA/AAc hydrogel without pH solution treatment needed to be sealed between a cleaned glass slide and a cover glass slide to store information. A three-dimensional piezostage (P-563.3CD, Physik Instrumente (PI) GmbH & Co. KG, Karlsruhe, Germany) was used to carry the sample at translational or axial displacements for the subsequent direct laser writing (DLW) experiment. Data entry was performed using a 532 nm femtosecond laser (210 fs, 80 MHz) tightly focused on the hydrogel through an oil-immersed objective lens (100×, NA = 1.4, Olympus, Tokyo, Japan). Raw data were recorded in the hydrogel via the sequential exposure of the fixed area in the hydrogel platform. A CCD camera (MER-132-43U3M-L, Daheng, Beijing, China) was used to monitor the data entry process.

### 2.5. Measurements and Characterizations

Scanning electron microscopy (SEM) was used to measure and characterize the degradable pH-responsive hydrogel. Before observation, the hydrogel was freeze-dried to remove all moisture, and then sputtered with a thin coating of gold. A dark-field microscope was used to extract scattering signals from plasmonic patterns embedded inside the hydrogel. The dark-field microscope system had a central wavelength of 595 nm of incident light with a 0.9 NA objective lens (MPlanFLN, 100×/0.9BD, Olympus) to provide a focal depth of 750 nm ($D.O.F.=\frac{n\lambda }{NA^{2}}+\frac{ne}{M\cdot NA}$) [39].

## 3. Results and Discussion

**Figure 1:**
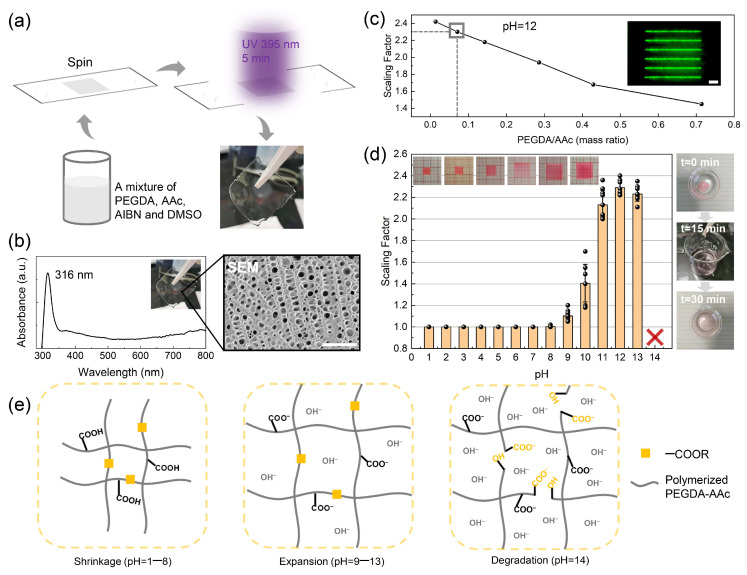
Preparation and characterization of the degradable pH-responsive hydrogel. (**a**) A schematic diagram of the preparation of hydrogel. (**b**) The left figure is the ultraviolet–visible (UV–Vis) absorption spectrum of the degradable pH-responsive hydrogel. The inset shows the degradable hydrogel photo. The right figure shows the SEM image of the hydrogel. The scale bar is 5 μm. (**c**) Effect of the PEGDA/AAc mass ratio on the scaling factor of the pH-responsive hydrogel at pH 12. The inset shows the dark-field image of a silver line array written in the degradable pH-responsive hydrogel with a component mass ratio of 0.071. The writing power is 0.3 mW and the writing speed is 3 μm/s. The scale bar is 5 μm. (**d**) pH response characteristics of the degradable pH-responsive hydrogel. The scaling factors at each pH value are statistically significant; the error bars represent the standard deviations of ten scaling factors tested at each pH value. When the pH values were 10 and 11, the stability of the scaling factor was poor. Illustrations show the hydrogels with different pH treatments. The right pictures show the degradation process of the gel in solution (pH 14, 50 °C) observed every 15 min. (**e**) Schematic diagram of three states of the hydrogel network at different pH values.

The degradable pH-responsive composite hydrogel provided a uniform environment for the stable photo-induced generation of a silver structure by the reactants inside the gel, which was an attractive and adjustable platform for preparing the silver structure. Furthermore, the hydrogel is rich in carboxylic acid groups which originate from AAc and the ester groups which originate from PEGDA [40]. The expanding behavior of the hydrogel is due to the deprotonation reaction in an alkaline environment [41] and the degradation of hydrogel mainly due to the hydrolysis of ester groups. During the deprotonation process initiated by hydroxide ions, the density of carboxylate ions (COO^−^) in the hydrogel network increases, and the hydrogel expands due to the volume phase transition caused by electrostatic repulsion forces. Firstly, a degradable pH-responsive hydrogel was prepared; a schematic diagram of the preparation of hydrogel is shown in Figure 1a. Meanwhile, the UV–Vis absorption spectrum of the hydrogel is shown with the maximum absorption peak at 316 nm in the left of Figure 1b; the inset shows a colorless and transparent hydrogel. The right of Figure 1b displays the SEM image of the degradable pH-responsive hydrogel. In order to achieve a larger scaling factor defined by Equation (1), the mass ratios of PEGDA to AAc have been adjusted, as shown in Figure 1c. As the mass ratio decreases, the scaling factor of the degradable hydrogel increases accordingly. When the PEGDA/AAc mass ratio was 0.014, the hydrogel was soft and not suitable for effectively supporting silver structures; therefore, we chose 0.071 as the PEGDA/AAc mass ratio, where PEGDA was 0.05 g, and AAc was 0.7 g. The inset shows a dark-field microscopic image of the silver line structures processed in the hydrogel with a 0.071 mass ratio of PEGDA/AAc.

In order to verify the pH response behavior of the PEGDA/AAc composite hydrogel, square pieces of PEGDA/AAc hydrogel with side lengths of 5 mm stored in deionized water were soaked in different pH solutions observed after 30 min at 50 °C. As shown in Figure 1d, hydrogels maintained a shrinkage state and the size barely changed when pH increased from 1.5 to 8, with a scaling factor of 1.0 corresponding to l = 5 mm. However, the hydrogels treated at higher pH values (pH = 9–13) were deformed significantly, with a scaling factor greater than 1, indicating the expansion of the hydrogel network. The threshold behavior of the degradable pH-responsive hydrogel can be attributed to the electrostatic effect caused by the carboxylic acid ionization of AAc monomers. As the pH value increased above 8, the hydroxide ions in the ambient fluid were sufficient to break the proton balance, provide the dominant repulsive force, and activate the expansion of the gel network. When the pH value reached 12 (corresponding to l = 11.5 mm), the carboxylic acid groups of AAc component were almost consumed and ionized. At the same time, the hydrogel reached the complete expansion state with the maximum scaling factor of 2.3. When the pH value increased up to 13, the scaling factor decreased slightly, which was related to the excessive hydroxide in the medium, so that the repulsion force between the ions inside and outside the network inhibited the further expansion of the gel network. The insets show that the hydrogel size changes corresponding to pH values increasing from 1.5 to 13. At a pH value of 14, excessive hydroxide ions in the surrounding environment of the hydrogel caused degradation of the hydrogel composition, PEGDA and AAc, leading to the collapse of the gelled matrix. At this time, PEGDA was decomposed into carboxylate and alcohol, and AAc was decomposed into sodium acrylate and water. Figure 1e shows three states of the hydrogel network at different pH values, corresponding to shrinkage (pH = 1–8), expansion (pH = 9–13) and degradation (pH = 14). The right figures in Figure 1d reflect the degradation process of the hydrogel in response to pH 14. The original morphology of the gel was a square piece; after soaking in pH = 14 for 15 min, the gel became soft with the filament shape shown in the middle image. The color of the soak solution changed from colorless to red, which indicated the release of R6G and laterally demonstrated the degradation progress of the degradable pH-responsive hydrogel.

As described in our strategy, prior to the decipherment process, the silver structures were recorded directly into the degradable pH-responsive hydrogel in a shrinkage state by the DLW technology, which did not cause any unwanted deformation of the gel morphology. The data were retrieved in the scattering of the sliver structures, and thus the effect of pH stimulation on the data was investigated. At first, the silver line array was recorded inside the original hydrogels, and then soaked together with the hydrogels in the configured solutions with different pH values for 30 min at 50 °C. The changes of the silver line array structure were observed by the dark-field imaging. It is worth mentioning that the scaling factor also applies to the line spacing of the silver line structures.

**Figure 2:**
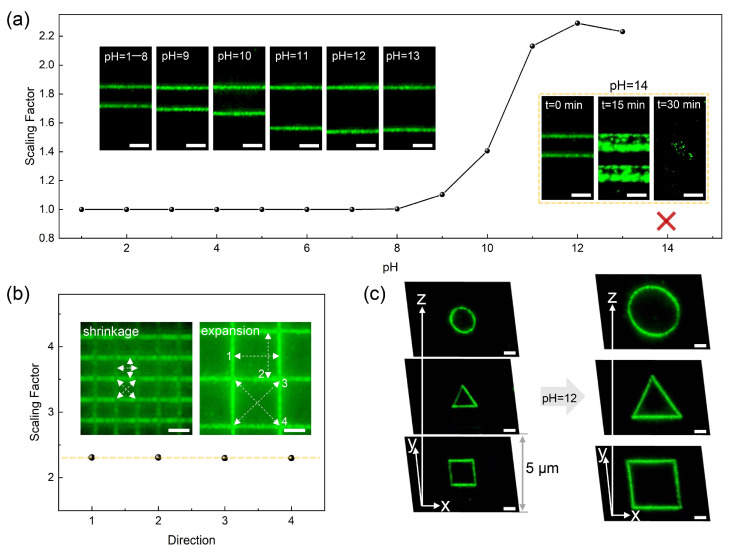
Dynamics of the recording information in the degradable pH-responsive hydrogel. (**a**) pH-responsive characteristics of the two silver line structures in the degradable hydrogel. The variations of the line spacing can also be represented by the scaling factor. The dark-field images of the silver line structures treated with different pH solutions are shown in the illustrations. The degradation processes of silver line structures are also shown in the illustrations with yellow dotted boxes (the sampling interval was 15 min). (**b**) Homogeneity test of platform scaling along four directions. The insets show the dark-field images of the silver grid structures in shrinkage states and full-expansion states. The original line spacings are 5 μm. White dotted arrows in the insets represent four diverse directions. (**c**) Dark-field images of a three-layer silver line pattern composed of the circular, triangular and square shapes in the hydrogel platform at initial or fully expanded state. The initial layer spacing is 5 μm. All the silver line patterns were recorded at a writing power of 0.3 mW and a writing speed of 3 μm/s. All scale bars are 5 μm.

Figure 2a reflects the scaling factors of silver line spacings along with the changes of pH, which is consistent with the scaling factor curve of the degradable pH-responsive hydrogel shown in Figure 1d. A good matching result revealed the synchronous changes of the gel and information, indicating that the silver particles reduced by the 532 nm fs laser were tightly adhering onto the gel. The scattering signals of silver line structures at pH values of 1.5–13 were read in the dark-field images shown in the illustrations. At pH 14, silver line structures gradually disappeared, accompanied by the degradation of hydrogel. Insets in the yellow dotted box are the dark-field images of the degradation process of silver line structures. When the immersing time accumulated to 15 min, silver line structures were distorted with the line width becoming coarse, which indicated that the pore structures of the gel had been degraded. After 30 min, silver line structures disappeared, and only a few free silver nanoparticles could be observed with the dark-field microscope. Meanwhile, homogeneity of the scaling process of hydrogel was also verified. As shown in Figure 2b, variations of the line spacings of the silver grid structures in four directions (white arrow) at the shrinkage state and the fully expansion state (pH of 12) were observed and measured by the dark-field imaging. Substituting the measured line spacings into Equation (1), the scaling factors in four directions were calculated and the negligible standard deviation was obtained, which showed that the degradable pH-responsive hydrogel possessed a superior expanding uniformity. Furthermore, we achieved the 3D information input in the degradable pH-responsive hydrogel as shown in Figure 2c. The left pictures are the dark-field images of three-layer data information, consisting of circular, triangular and square silver structures with a vertical layer spacing of 5 μm in hydrogel with a shrinkage state. The right pictures are the dark-field images of the three-layer silver line structures within the hydrogel platform of the equilibrium state (pH 12). Compared with the original silver pattern, the sizes of the 3D silver pattern treated with solution of pH 12 increased and the layer spacings changed accordingly.

It is well known that the focal depth of an imaging system is given by the formula $D.O.F.=\frac{n\lambda }{NA^{2}}+\frac{ne}{M\cdot NA}$ [35] (where *n* is the refractive index of the object area, *λ* is the wavelength of incident light, *NA* is the numerical aperture of the microscope objective, *e* represents the minimum distance that the human eye can distinguish, and M is the magnification of the viewing angle). Due to the diffraction of light, two objects spaced less than the *D.O.F.* of the detection system are difficult to distinguish. In our study, with the help of high-precision DLW technology, the vertical spacings of recorded structures were set below the *D.O.F.* value of the detection microscope system, so that the expanding behavior of the hydrogel platform could drive the vertical space beyond the focal depth. Therefore, if the vertical spacings of recorded structures were less than the focal depth of the detection system, the crosstalk between the structures could distort the original information and fail to decode the acquired signal.

**Figure 3:**
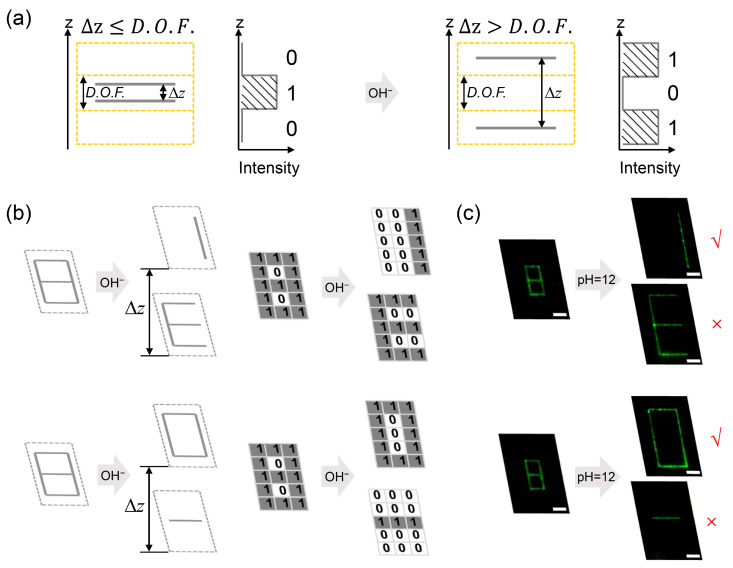
Model and experimental verification for the 3D optical data encryption method. (**a**) The encryption schematic diagram and coded diagram. The vertical spacing of two silver lines is denoted by ∆*z*, and the lengths of yellow dotted line units in the z direction denotes the size of focal depth of the detection system. The initial vertical spacing of two silver lines shown in the left figure is less than the focal depth of the detection system. Additionally, only the middle unit area can emit the scattering signal. The figure on the right shows that the line spacing is larger than the focal depth of the detection system with a specific alkaline stimulus. In this case, line structures occupied two focal depth units. The focal depth unit with the scattering signal is encoded as “1”; otherwise, they are encoded as “0”. (**b**) The two upper figures indicate the encryption schematic diagram and coded diagram of the effective information “1”. The two following figures are the encryption schematic diagram and coded diagram of the effective information “0”. ∆*z* is greater than the focal depth of the detection system. (**c**) The experimental dark-field images of the effective digital information “1” and “0” correspond to the schematic model in (**b**). All silver line structures were fabricated at the written conditions of 0.1 mW, 3 μm/s. All scale bars are 5 μm.

As a proof-of-concept demonstration, a specific two-layer silver line array was designed, as exhibited in Figure 3a. It is seen that the two-layer silver line array consisted of two silver nanolines with the vertical spacing, ∆*z*, spaced less than the focal depth of the dark-field microscopic system. The vertical space was divided into several vertical units, shown as the yellow dotted grid, by the size of focal depth of the detection system. When the vertical line spacing was less than the focal depth, the space occupied by two silver lines was compressed within a focal depth unit. Then, the microscope system read out the overlapping information consisting of the two silver-line scattering signals. The vertical spacing of two silver nanolines would be larger than the focal depth if the carrier hydrogel was in the expansion state. At this time, two sliver nanolines occupying different voxels can be read. Figure 3a also presents the code definition. The code of voxel delivering the silver scattering signal is defined as “1”. Conversely, the code of voxel without silver scattering signal is defined as “0”. Hence, any silver scattering information can be replaced by the specific coded array. Corresponding to the structured voxels in the schematic model diagram, the information code array in the vertical direction changes from “010” to “101” after dealing with the specific alkaline pH solution. Figure 3b shows the designed decryption model of different effective information, with numbers “1” and “0” shown here. The initial input information consisted of a double-layer silver line array in which one layer was the effective information layer and the other was the interference information layer with layer-to-layer spacing Δz≤D.O.F. In three-dimensional space, a single voxel in hydrogel giving rise to the light scattering signal is regarded as the recording pixel “1”, whose side lengths in the x–y and z direction correspond to the horizontal and the vertical resolution of the decoding system, respectively, while the voxel without scattering signals is recoded as “0”. Therefore, the scattering signals from the silver plasmonic structures can be converted into binary data patterns composed of “0” and “1”. Due to the layer spacing of the structured silver voxels being less than the depth of focus, a corresponding single-layer coded array is obtained as shown on the right. After decryption, the layer spacing of the silver structures is larger than the depth of focus (Δz>D.O.F.); a corresponding double-layer coded array is obtained, as shown on the right. Figure 3c shows the dark-field microscopic images obtained in the experiment. Taking the effective information “1” as an example: first, the Arabic number “1” and pattern “E” were recorded in the degradable pH-responsive hydrogel in a shrinkage state by positioning the ultra-fast laser beam along the predetermined trajectory with a laser power of 0.1 mW and a recording speed of 3 μm/s. The layer spacing between the two silver structures was set at 750 nm, as shown in Figure 3b (upper left). Additionally, a dark-field microscope with a vertical focal depth of 750 nm (*λ* = 595 nm, *NA* = 0.9) was used to collect the scattering signals of the double-layer silver pattern. Then, we immersed the hydrogel in NaOH solution with a pH of 12 for 30 min at a temperature of 50 °C, and thus obtained the effective information, “1”. In this way, the feasibility of this encryption method was proved experimentally.

**Figure 4:**
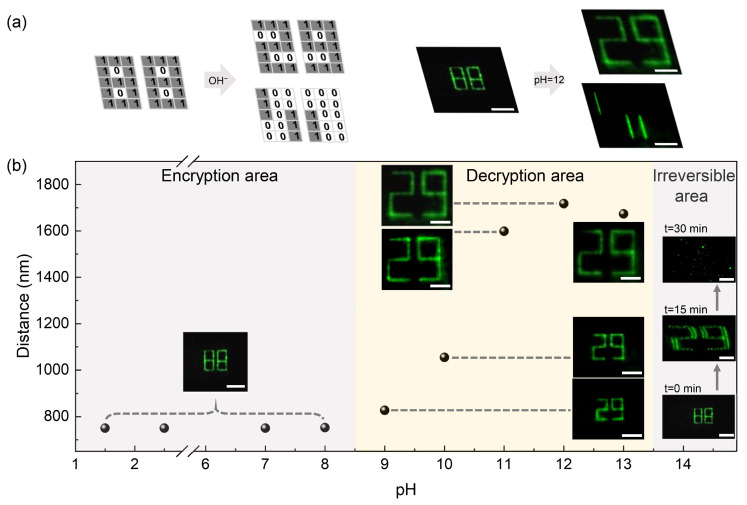
The secure 3D data encryption strategy. (**a**) Encryption coded diagrams of effective sequence information “29” (left) and the corresponding experimental dark-field diagrams (right). (**b**) Effect of pH on information in the degradable pH-responsive hydrogel. The illustrations are the dark-field images of silver line structures soaked in different pH solutions for 30 min at 50 °C. The encryption area is at pH 1–8, the decryption area is at pH 9–13, and the irreversible area is at pH 14, which shows the information collapse process. The writing power of silver nanostructures is 0.1 mW and the writing speed is 3 μm/s. All scale bars are 10 μm.

In order to highlight the security characteristics of the data encryption strategy, three-dimensional encryption of the digital sequence “29” is presented in Figure 4. The encoded diagram is shown on the left of Figure 4a. At first, the effective information of the digital pattern “29” was recorded by patterned code “1” array, and the interference information containing three vertical lines was embedded into the hydrogel in a shrinkage state. Spacing between the effective information layer and the interference information layer was precisely set as the focal depth of the dark-field microscope system, which was 750 nm. In this case, the crosstalk of the scattering signals of two-layer silver nanoline structures led to the distortion of effective information; we could only read out the digital information “88” composed of the code “1” array for misinformation. However, the digital information “29” could be read out when the hydrogel platform was in an expansion state. Meanwhile, the layer spacing between the two silver patterns was larger than 750 nm. In the experiment, a 0.1 mW writing power and 3 μm/s writing speed were used to input the silver nanostructures into the degradable pH-responsive hydrogel. Then, the hydrogel platform was treated with an alkaline solution of pH 12, and the effective digital information “29” could be read out with the expansion of the gel network.

Figure 4b shows the changes of the digital information treated with different pH keys. The abscissa represents the pH value, and the ordinate represents the vertical layer spacing of the silver patterns. As discussed above, the degradable pH-responsive hydrogels were prepared in advance, and two layers of silver nanoline digital patterns with a spacing of 750 nm were recorded under the same writing conditions (0.1 mW, 3 μm/s). Then, the recorded hydrogels were immersed in pH 1.5–14 solutions for 30 min at 50 °C and observed through a dark-field microscope. As can be seen, the whole range of pH keys is divided into three limited windows, namely, the encryption area, decryption area and irreversible sabotage area. In the encryption area, internal digital information treated with pH values from 1.5 to pH 8 was consistent with that of the non-pH-treated information, showing “88”. In the decryption area, with pH values between 9 and 13, the internal effective digital information could be decoded and displayed as “29” with the hydrogel in an expansion state. The irreversible area was at pH 14, where the excessive hydroxyl groups in the environment led to the input digital information disappearing with the collapse of the hydrogel network. Therefore, this method can not only retrieve the information with an effective key, but also destroy the complete information with an invalid key by using an irreversible chemical reaction.

## 4. Conclusions

In summary, we have developed a security-enhanced 3D data encryption technique by using degradable pH-responsive hydrogel for enhancing information security. The degradable pH-responsive hydrogel was prepared by using a PEGDA/AAc composite. It was found that the hydrogel exhibited shrinkage, expansion or degradation states under the different pH stimulations, giving rise to two pH value thresholds for state transitions. The responses of gel matrix scaling could only be triggered at specific pH regions. Benefitting from the expansion threshold, external pH values of 9–13 were used as the decryption key to obtain the effective information, whereas an invalid pH value above 14 could damage the effective data owing to pH values exceeding the threshold of hydrogel degradation. In our study, because external pH stimulation initiated the scaling behavior of the degradable pH-responsive hydrogel, the vertical layer spacing of the encoded structures could be modulated within/outside the depth of focus region of the detection system. By precisely matching the vertical focal depth of the optical decryption system and the scaling factor of the hydrogel, a customizable 3D silver structured voxel array was designed to store the effective scattering information. The results show that it is feasible to achieve the optical decryption of custom 3D patterns without significant signal distortion, and the encrypted message can be read correctly only at the appropriate pH region as the key for information decryption. The strategy represents a highly secure 3D data encoding protocol and can open the way for developing novel chemical channels to protect vital information.

## Data Availability

The data presented in this article are available on request from the corresponding author.
